# Supplementary material for: Transcriptomic Analysis of the Differential Nephrotoxicity of Diverse Brominated Flame Retardants in Rat and Human Renal Cells
Source: Int J Mol Sci. 2021 Sep 17;22(18):10044. doi: 10.3390/ijms221810044 (PMC8465879; doi:10.3390/ijms221810044)
Supplement: Supplementary file 1 [file ijms-22-10044-s001.zip › ijms-1288438-supplementary.pdf]

Table S1. Differentially expressed genes in HK-2 cells after BDE-47 exposure

Gene ID	Gene Name	Q value	P value	Log FC
ENSG00000268220	AC008040.5	0.034637	0.002474	1.077264
ENSG00000273489	AC008264.2	0.000241	2.60E-06	2.568728
ENSG00000269242	AC010422.3	0.008163	0.000268	2.262132
ENSG00000279592	AC010653.2	0.00495	0.000134	1.344836
ENSG00000255498	AC068385.1	0.01964	0.001052	1.931164
ENSG00000282033	AC074387.1	0.012087	0.000486	1.071099
ENSG00000220793	AC087190.1	0.000282	3.24E-06	1.193149
ENSG00000254665	AC091053.1	0.046613	0.003978	2.27546
ENSG00000271787	AC104794.4	0.005169	0.000142	1.915886
ENSG00000219410	AC125494.1	0.047298	0.004076	1.223314
ENSG00000102575	ACP5	1.21E-07	3.10E-10	1.203135
ENSG00000154734	ADAMTS1	1.48E-08	2.86E-11	-1.07425
ENSG00000261641	AL031600.3	0.000597	7.89E-06	1.77265
ENSG00000238164	AL139246.5	6.69E-09	1.20E-11	1.584169
ENSG00000225339	AL354740.1	0.004552	0.00012	3.63711
ENSG00000284128	AP000356.3	0.00012	1.10E-06	1.230886
ENSG00000254873	AP001267.1	0.021045	0.001169	1.948798
ENSG00000130203	APOE	0.036613	0.002701	1.076347
ENSG00000100628	ASB2	0.009019	0.000311	1.356537
ENSG00000147614	ATP6V0D2	0.000101	9.05E-07	1.101865
ENSG00000101197	BIRC7	1.61E-08	3.23E-11	1.688676
ENSG00000182795	C1orf116	0.000741	1.01E-05	-1.00611
ENSG00000074410	CA12	3.19E-06	1.26E-08	1.017948
ENSG00000012124	CD22	2.10E-06	7.14E-09	1.016879
ENSG00000138395	CDK15	0.003408	7.88E-05	-1.68988
ENSG00000239704	CDRT4	0.045028	0.003799	1.146551
ENSG00000103196	CRISPLD2	0.000194	2.04E-06	-1.24298
ENSG00000103316	CRYM	0.000316	3.70E-06	1.184108
ENSG00000172346	CSDC2	1.17E-06	3.82E-09	1.311166
ENSG00000142871	CYR61	9.98E-14	6.91E-17	-1.00995
ENSG00000073737	DHRS9	0.000322	3.81E-06	1.186376
ENSG00000115380	EFEMP1	0.000583	7.67E-06	-1.05567
ENSG00000156804	FBXO32	1.34E-21	9.24E-26	1.285656
ENSG00000158869	FCER1G	0.048857	0.004267	1.423952
ENSG00000265817	FSBP	0.023236	0.001349	1.482972
ENSG00000130222	GADD45G	0.00879	0.000297	1.06535
ENSG00000155265	GOLGA7B	6.21E-06	2.66E-08	1.03965
ENSG00000136235	GPNMB	4.13E-14	2.57E-17	1.140957
ENSG00000161835	GRASP	8.47E-06	3.99E-08	1.071031
ENSG00000244067	GSTA2	0.000264	2.94E-06	1.740084
ENSG00000242574	HLA-DMB	1.51E-14	8.37E-18	1.130248
ENSG00000105707	HPN	0.002072	4.22E-05	1.104789
ENSG00000101384	JAG1	2.45E-07	6.49E-10	1.141582
ENSG00000135253	KCP	2.12E-08	4.54E-11	1.585708
ENSG00000212724	KRTAP2-3	0.000177	1.79E-06	-1.18082
ENSG00000187416	LHFPL3	0.004214	0.000106	1.797539
ENSG00000225329	LHFPL3-AS2	0.005697	0.000164	1.462097

ENSG00000185186	LINC00313	0.002852	6.24E-05	1.159082
ENSG00000231776	LINC01611	0.026084	0.001612	1.041742
ENSG00000100253	MIOX	2.29E-20	3.17E-24	1.495341
ENSG00000067798	NAV3	1.03E-06	3.21E-09	-1.05255
ENSG00000169418	NPR1	1.28E-14	6.23E-18	1.184375
ENSG00000012504	NR1H4	0.005803	0.000171	1.284558
ENSG00000176046	NUPR1	0.000657	8.82E-06	1.558888
ENSG00000004799	PDK4	1.41E-05	8.01E-08	1.025633
ENSG00000135549	PKIB	3.67E-09	5.84E-12	1.080784
ENSG00000104368	PLAT	1.30E-18	2.70E-22	-1.05259
ENSG00000100033	PRODH	6.47E-05	5.28E-07	1.183644
ENSG00000102032	RENBP	2.20E-05	1.39E-07	1.7467
ENSG00000230207	RPL4P5	0.010343	0.000383	1.051224
ENSG00000135094	SDS	0.000727	9.87E-06	1.268985
ENSG00000138623	SEMA7A	1.14E-13	8.71E-17	-1.00814
ENSG00000106366	SERPINE1	4.08E-17	1.13E-20	-1.31538
ENSG00000167711	SERPINF2	0.019239	0.001018	1.031799
ENSG00000143554	SLC27A3	8.05E-06	3.57E-08	1.192682
ENSG00000095932	SMIM24	1.73E-05	1.04E-07	1.107406
ENSG00000188176	SMTNL2	6.17E-11	5.94E-14	2.118032
ENSG00000164266	SPINK1	0.014351	0.000659	1.435922
ENSG00000182557	SPNS3	0.001979	3.89E-05	1.066972
ENSG00000164744	SUN3	0.042812	0.003448	-1.00284
ENSG00000170006	TMEM154	8.12E-12	6.75E-15	-1.03459
ENSG00000206140	TMEM191C	0.001442	2.56E-05	1.019586
ENSG00000171227	TMEM37	8.68E-06	4.20E-08	1.087179
ENSG00000157873	TNFRSF14	2.90E-16	1.01E-19	1.187668
ENSG00000187688	TRPV2	0.002033	4.09E-05	1.333955
ENSG00000186919	ZACN	0.025385	0.001533	5.091299
ENSG00000178150	ZNF114	8.10E-15	3.36E-18	1.358482

Table S2. Differentially expressed genes in HK-2 cells after HBCD exposure

Gene ID	Gene Name	Q value	P value	Log FC
ENSG00000273489	AC008264.2	0.016594	0.000207	1.989328
ENSG00000270607	AC009549.1	7.13E-06	1.04E-08	1.126095
ENSG00000279592	AC010653.2	0.026738	0.000413	1.233077
ENSG00000238164	AL139246.5	0.0018	7.13E-06	1.04669
ENSG00000225339	AL354740.1	0.020784	0.000286	3.400935
ENSG00000167772	ANGPTL4	0.000798	2.60E-06	1.093564
ENSG00000140450	ARRDC4	1.64E-07	1.14E-10	-1.04822
ENSG00000100628	ASB2	0.006165	4.95E-05	1.525706
ENSG00000147614	ATP6V0D2	1.91E-09	6.60E-13	1.576584
ENSG00000074410	CA12	1.01E-05	1.54E-08	1.0112
ENSG00000088340	FER1L4	9.00E-08	4.98E-11	1.070898
ENSG00000265817	FSBP	0.016616	0.000209	1.711906
ENSG00000128242	GAL3ST1	0.004337	3.06E-05	1.180107
ENSG00000130513	GDF15	6.67E-06	9.24E-09	1.079559
ENSG00000242574	HLA-DMB	8.64E-14	1.20E-17	1.122345
ENSG00000135253	KCP	1.55E-05	2.69E-08	1.332589
ENSG00000134545	KLRC1	0.022863	0.000331	1.100211
ENSG00000185186	LINC00313	0.003526	2.12E-05	1.215463
ENSG00000247095	MIR210HG	8.07E-05	1.77E-07	1.188444
ENSG00000104419	NDRG1	8.64E-14	1.10E-17	1.245054
ENSG00000176046	NUPR1	0.007664	7.11E-05	1.38117
ENSG00000188176	SMTNL2	4.64E-05	9.64E-08	1.509104
ENSG00000265972	TXNIP	3.73E-07	3.36E-10	-1.2534
ENSG00000214049	UCA1	0.011048	0.000119	1.053742
ENSG00000178150	ZNF114	3.45E-11	9.56E-15	1.203731

Table S3. Differentially expressed genes in HK-2 cells after TBBPA exposure				
Gene ID	Gene Name	Q value	P value	Log FC
ENSG00000235852	AC005540.1	0.003676	0.000391	-1.59389
ENSG00000262185	AC005736.1	0.000154	8.16E-06	1.24623
ENSG00000262888	AC005736.2	0.013894	0.002072	1.002454
ENSG00000267469	AC005944.1	0.040632	0.008639	1.006333
ENSG00000268220	AC008040.5	0.015159	0.002323	1.084926
ENSG00000273489	AC008264.2	0.00039	2.55E-05	2.27858
ENSG00000279592	AC010653.2	0.011709	0.001673	1.115757
ENSG00000261777	AC012184.3	0.004125	0.000451	1.048673
ENSG00000266872	AC015688.8	0.014869	0.002258	1.822319
ENSG00000274213	AC015912.3	0.006418	0.000782	1.0854
ENSG00000272831	AC027644.3	0.002888	0.000292	1.026608
ENSG00000225205	AC078883.1	2.75E-06	8.03E-08	-1.79455
ENSG00000279033	AC090984.1	0.023633	0.004191	1.926385
ENSG00000223396	AC096677.1	1.90E-07	3.82E-09	1.351556
ENSG00000253882	AC099548.2	1.38E-06	3.66E-08	-1.61925
ENSG00000271787	AC104794.4	0.00298	0.000304	1.818109
ENSG00000261298	AC122134.1	0.010831	0.001507	-1.07948
ENSG00000223804	AC244669.1	8.77E-06	2.98E-07	1.073745
ENSG00000181790	ADGRB1	0.00054	3.69E-05	1.335251
ENSG00000153292	ADGRF1	1.30E-05	4.78E-07	-1.67041
ENSG00000173698	ADGRG2	6.87E-06	2.26E-07	-1.61686
ENSG00000111863	ADTRP	2.45E-07	5.14E-09	1.156573
ENSG00000154027	AK5	3.37E-11	3.19E-13	1.075193
ENSG00000261641	AL031600.3	0.013841	0.002061	1.210721
ENSG00000235142	AL080314.2	4.39E-06	1.36E-07	-2.06142
ENSG00000238164	AL139246.5	0.000107	5.36E-06	1.071165
ENSG00000225339	AL354740.1	0.016216	0.002544	2.781692
ENSG00000228748	AL450306.1	0.032651	0.006454	1.166826
ENSG00000229953	AL590666.2	0.000322	2.01E-05	1.052138
ENSG00000136010	ALDH1L2	1.71E-11	1.52E-13	1.297118
ENSG00000132965	ALOX5AP	0.010831	0.001506	1.173334
ENSG00000254873	AP001267.1	0.010218	0.001396	1.916608
ENSG00000109321	AREG	3.09E-06	9.12E-08	1.074582
ENSG00000081181	ARG2	8.42E-23	1.34E-25	1.29215
ENSG00000116584	ARHGEF2	1.51E-21	2.61E-24	1.100003
ENSG00000070669	ASNS	2.10E-40	5.83E-44	1.486603
ENSG00000162772	ATF3	8.78E-11	8.69E-13	1.180838
ENSG00000228218	ATF4P3	0.007053	0.000876	1.005207
ENSG00000147614	ATP6V0D2	1.87E-42	2.59E-46	3.152642
ENSG00000105327	BBC3	8.51E-08	1.59E-09	1.185476
ENSG00000133134	BEX2	2.42E-29	1.68E-32	2.635529

ENSG00000023445	BIRC3	2.63E-17	9.84E-20	-1.11152
ENSG00000273145	BX537318.1	0.00163	0.000141	1.021242
ENSG00000273812	BX640514.2	3.01E-07	6.56E-09	1.274678
ENSG00000165507	C10orf10	3.45E-10	3.74E-12	-1.32361
ENSG00000214212	C19orf38	0.000569	3.94E-05	1.299919
ENSG00000180044	C3orf80	1.84E-07	3.66E-09	1.236065
ENSG00000197405	C5AR1	5.88E-12	4.93E-14	1.890318
ENSG00000204387	C6orf48	3.05E-14	1.67E-16	1.094357
ENSG00000164746	C7orf57	4.98E-10	5.52E-12	1.371477
ENSG00000172137	CALB2	8.33E-09	1.20E-10	1.208488
ENSG00000272398	CD24	1.09E-15	5.12E-18	-1.39098
ENSG00000166589	CDH16	3.30E-05	1.41E-06	-1.15571
ENSG00000113361	CDH6	2.60E-16	1.12E-18	-1.97086
ENSG00000124762	CDKN1A	1.43E-18	4.74E-21	1.661858
ENSG00000239704	CDRT4	0.00189	0.000169	1.499596
ENSG00000172216	CEBPB	2.71E-07	5.75E-09	1.007701
ENSG00000128965	CHAC1	1.53E-19	4.02E-22	1.370771
ENSG00000165376	CLDN2	3.54E-19	1.05E-21	-2.07261
ENSG00000069493	CLEC2D	4.32E-15	2.13E-17	1.332466
ENSG00000143786	CNIH3	9.16E-17	3.68E-19	1.463659
ENSG00000128510	CPA4	5.21E-09	7.03E-11	-1.26461
ENSG00000168993	CPLX1	1.28E-05	4.68E-07	1.105486
ENSG00000176092	CRYBG2	5.96E-06	1.92E-07	1.224926
ENSG00000150527	CTAGE5	1.28E-23	1.60E-26	1.033078
ENSG00000116761	CTH	5.76E-69	3.99E-73	1.792338
ENSG00000081041	CXCL2	8.02E-08	1.48E-09	1.247454
ENSG00000163734	CXCL3	2.98E-05	1.26E-06	1.15543
ENSG00000169429	CXCL8	0.002145	0.000199	1.037395
ENSG00000138061	CYP1B1	0.000349	2.22E-05	1.040718
ENSG00000175197	DDIT3	9.42E-20	2.41E-22	1.907917
ENSG00000164825	DEFB1	3.53E-06	1.06E-07	-1.20543
ENSG00000099958	DERL3	2.34E-07	4.88E-09	1.011703
ENSG00000107984	DKK1	9.95E-05	4.93E-06	-1.38225
ENSG00000262001	DLGAP1-AS2	5.41E-09	7.45E-11	1.073424
ENSG00000128590	DNAJB9	1.28E-23	1.68E-26	1.376668
ENSG00000149599	DUSP15	1.23E-06	3.17E-08	1.780887
ENSG00000129521	EGLN3	2.05E-11	1.83E-13	-1.44223
ENSG00000154928	EPHB1	4.97E-08	8.64E-10	1.016228
ENSG00000178607	ERN1	2.38E-24	2.63E-27	1.128521
ENSG00000135842	FAM129A	7.15E-16	3.22E-18	1.095722
ENSG00000185112	FAM43A	2.08E-06	5.79E-08	1.54436
ENSG00000173295	FAM86B3P	5.43E-05	2.48E-06	1.242144
ENSG00000214050	FBXO16	6.95E-06	2.29E-07	1.10716

ENSG00000161243	FBXO27	1.46E-05	5.47E-07	1.001023
ENSG00000158869	FCER1G	0.027069	0.005029	1.400898
ENSG00000272143	FGF14-AS2	4.57E-05	2.04E-06	1.137791
ENSG00000066468	FGFR2	0.000361	2.32E-05	-1.75119
ENSG00000125848	FLRT3	0.006707	0.000826	-1.01442
ENSG00000114541	FRMD4B	3.23E-18	1.09E-20	-1.29288
ENSG00000265817	FSBP	9.55E-05	4.67E-06	2.138645
ENSG00000226608	FTLP3	0.003104	0.00032	1.068521
ENSG00000116717	GADD45A	2.86E-14	1.52E-16	1.179885
ENSG00000130222	GADD45G	0.002157	0.000202	1.09236
ENSG00000182870	GALNT9	0.000208	1.18E-05	1.818153
ENSG00000234741	GAS5	2.10E-17	7.70E-20	1.19786
ENSG00000130513	GDF15	1.01E-18	3.29E-21	1.810615
ENSG00000131459	GFPT2	3.32E-13	2.27E-15	1.776097
ENSG00000155265	GOLGA7B	1.55E-09	1.93E-11	1.257797
ENSG00000125772	GPCPD1	6.88E-12	5.86E-14	1.003138
ENSG00000164849	GPR146	0.015105	0.002308	-1.17028
ENSG00000237310	GS1-124K5.4	0.000382	2.49E-05	1.121429
ENSG00000136732	GYPC	4.78E-13	3.37E-15	1.113178
ENSG00000130600	H19	0.00011	5.52E-06	-2.07663
ENSG00000051108	HERPUD1	3.32E-14	1.89E-16	1.01747
ENSG00000144485	HES6	0.000159	8.49E-06	1.040094
ENSG00000100292	HMOX1	2.10E-40	4.36E-44	2.622827
ENSG00000164120	HPGD	8.66E-19	2.76E-21	-1.07807
ENSG00000044574	HSPA5	1.06E-10	1.07E-12	1.060411
ENSG00000003147	ICA1	3.60E-11	3.46E-13	1.056381
ENSG00000244242	IFITM10	3.35E-05	1.44E-06	1.292889
ENSG00000172458	IL17D	0.000223	1.28E-05	1.017891
ENSG00000103522	IL21R	5.98E-07	1.43E-08	1.514887
ENSG00000110944	IL23A	1.59E-05	6.10E-07	1.097003
ENSG00000139269	INHBE	2.03E-16	8.44E-19	1.73033
ENSG00000137825	ITPKA	3.83E-11	3.71E-13	1.704644
ENSG00000026559	KCNG1	5.85E-07	1.39E-08	1.667645
ENSG00000135253	KCP	2.25E-06	6.37E-08	1.308266
ENSG00000049130	KITLG	2.32E-19	6.59E-22	-1.62708
ENSG00000134545	KLRC1	0.000661	4.75E-05	1.256032
ENSG00000166173	LARP6	2.45E-14	1.27E-16	1.071563
ENSG00000213658	LAT	1.55E-10	1.60E-12	1.024837
ENSG00000134333	LDHA	1.86E-19	5.16E-22	-1.07889
ENSG00000214110	LDHAP4	0.000644	4.60E-05	-1.12196
ENSG00000187416	LHFPL3	0.005024	0.000577	-1.76553
ENSG00000225329	LHFPL3-AS2	0.001664	0.000145	-1.52938
ENSG00000185186	LINC00313	0.001396	0.000115	1.12279

ENSG00000225511	LINC00475	0.001115	8.77E-05	1.060495
ENSG00000274020	LINC01138	1.04E-09	1.25E-11	1.256711
ENSG00000180861	LINC01559	2.46E-19	7.16E-22	-2.09988
ENSG00000166407	LMO1	7.05E-07	1.71E-08	2.054636
ENSG00000171236	LRG1	2.37E-08	3.80E-10	-1.13708
ENSG00000125872	LRRN4	1.08E-13	6.60E-16	-1.19527
ENSG00000049323	LTBP1	4.28E-13	2.96E-15	1.169709
ENSG00000153714	LURAP1L	5.31E-26	4.78E-29	2.072092
ENSG00000095015	MAP3K1	2.65E-11	2.44E-13	-1.22859
ENSG00000271605	MILR1	1.14E-07	2.17E-09	1.314614
ENSG00000247095	MIR210HG	1.20E-07	2.30E-09	-1.46393
ENSG00000150051	MKX	3.12E-11	2.91E-13	1.334642
ENSG00000137673	MMP7	8.06E-10	9.43E-12	-1.88326
ENSG00000178860	MSC	2.32E-08	3.70E-10	1.378755
ENSG00000185499	MUC1	4.06E-08	6.85E-10	-1.0908
ENSG00000007944	MYLIP	1.02E-12	7.60E-15	-1.11073
ENSG00000198496	NBR2	3.09E-12	2.53E-14	1.515243
ENSG00000103154	NECAB2	9.90E-07	2.49E-08	1.765967
ENSG00000111859	NEDD9	6.74E-18	2.33E-20	-1.2169
ENSG00000107954	NEURL1	8.06E-10	9.41E-12	1.334089
ENSG00000135540	NHSL1	4.49E-09	6.00E-11	1.003683
ENSG00000012504	NR1H4	0.001066	8.32E-05	-1.56355
ENSG00000205309	NT5M	0.000278	1.68E-05	1.025857
ENSG00000176046	NUPR1	8.23E-12	7.06E-14	2.59416
ENSG00000101104	PABPC1L	7.27E-13	5.29E-15	1.202038
ENSG00000116183	PAPPA2	1.59E-08	2.48E-10	1.561962
ENSG00000100889	PCK2	1.64E-37	5.67E-41	1.398512
ENSG00000186642	PDE2A	3.78E-05	1.64E-06	1.130158
ENSG00000234465	PINLYP	0.00054	3.69E-05	1.003117
ENSG00000204531	POU5F1	2.05E-07	4.17E-09	-1.15065
ENSG00000143847	PPFIA4	1.14E-08	1.67E-10	-1.64565
ENSG00000087074	PPP1R15A	4.18E-25	4.05E-28	1.377863
ENSG00000135406	PRPH	3.42E-17	1.30E-19	2.598638
ENSG00000135069	PSAT1	4.68E-29	3.56E-32	1.077904
ENSG00000248643	RBM14-RBM4	0.000244	1.44E-05	-1.25104
ENSG00000102032	RENBP	0.010667	0.001476	1.065179
ENSG00000071242	RPS6KA2	3.01E-12	2.43E-14	1.351461
ENSG00000166922	SCG5	0.000248	1.46E-05	1.30884
ENSG00000139410	SDSL	1.19E-10	1.22E-12	1.152259
ENSG00000170099	SERPINA6	3.35E-07	7.50E-09	-1.22218
ENSG00000130766	SESN2	2.49E-24	2.93E-27	1.130695
ENSG00000145423	SFRP2	0.000134	7.01E-06	1.689564
ENSG00000118515	SGK1	2.57E-28	2.13E-31	-1.51328

ENSG00000125089	SH3TC1	5.82E-08	1.05E-09	1.014884
ENSG00000168679	SLC16A4	1.78E-12	1.38E-14	1.057185
ENSG00000108932	SLC16A6	5.08E-08	8.94E-10	1.900172
ENSG00000165449	SLC16A9	1.14E-05	4.10E-07	1.000722
ENSG00000163393	SLC22A15	3.14E-14	1.74E-16	1.204282
ENSG00000163581	SLC2A2	3.13E-13	2.12E-15	-1.73594
ENSG00000134955	SLC37A2	1.88E-06	5.20E-08	1.181495
ENSG00000168003	SLC3A2	4.09E-15	1.98E-17	1.128674
ENSG00000149150	SLC43A1	2.64E-20	5.90E-23	1.674306
ENSG00000050438	SLC4A8	3.17E-11	2.99E-13	-1.18146
ENSG00000196517	SLC6A9	1.85E-21	3.59E-24	1.730616
ENSG00000151012	SLC7A11	8.52E-30	5.31E-33	1.863244
ENSG00000103257	SLC7A5	6.79E-14	4.09E-16	1.043127
ENSG00000166750	SLFN5	6.90E-13	4.97E-15	1.006732
ENSG00000261556	SMG1P7	7.71E-08	1.42E-09	1.274298
ENSG00000178947	SMIM10L2A	1.22E-09	1.49E-11	1.40811
ENSG00000240204	SMKR1	0.00628	0.000762	1.174075
ENSG00000120833	SOCS2	1.21E-05	4.38E-07	1.29662
ENSG00000134548	SPX	2.31E-07	4.79E-09	1.45914
ENSG00000198203	SULT1C2	6.46E-20	1.57E-22	1.007914
ENSG00000142765	SYTL1	2.92E-07	6.32E-09	1.37677
ENSG00000178726	THBD	9.48E-37	3.94E-40	2.767372
ENSG00000185561	TLCD2	1.23E-12	9.33E-15	1.152916
ENSG00000145107	TM4SF19	3.04E-14	1.64E-16	1.084458
ENSG00000136404	TM6SF1	1.54E-14	7.80E-17	2.407914
ENSG00000127863	TNFRSF19	1.05E-12	7.89E-15	-1.09601
ENSG00000121858	TNFSF10	4.66E-19	1.42E-21	-1.30068
ENSG00000124191	TOX2	1.44E-24	1.49E-27	1.041135
ENSG00000101255	TRIB3	2.07E-23	3.01E-26	1.554167
ENSG00000178809	TRIM73	0.015121	0.002313	1.008155
ENSG00000214049	UCA1	1.25E-06	3.24E-08	-1.57285
ENSG00000111981	ULBP1	8.13E-09	1.15E-10	1.433423
ENSG00000183696	UPP1	8.59E-31	4.16E-34	2.212282
ENSG00000134215	VAV3	3.01E-12	2.42E-14	-1.0476
ENSG00000038427	VCAN	7.66E-07	1.88E-08	-1.06419
ENSG00000128564	VGF	1.26E-20	2.63E-23	1.627162
ENSG00000236404	VLDLR-AS1	8.33E-09	1.20E-10	1.767913
ENSG00000070540	WIPI1	2.35E-30	1.30E-33	1.256669
ENSG00000186919	ZACN	0.030058	0.005786	4.265704
ENSG00000104231	ZFAND1	1.52E-21	2.73E-24	1.048382
ENSG00000178381	ZFAND2A	1.04E-15	4.83E-18	1.095001
ENSG00000177410	ZFAS1	5.91E-16	2.62E-18	1.138104
ENSG00000251003	ZFPM2-AS1	1.01E-08	1.48E-10	1.067106

ENSG00000224596	ZMIZ1-AS1	1.84E-15	8.77E-18	1.419135

Table S4. Differentially expressed genes in NRK cells after BDE-47 exposure

Gene ID	Gene Name	Q value	P value	Log FC
ENSRNOG00000018126	Abca1	1.04E-13	4.48E-17	-1.18801376
ENSRNOG00000027742	Adamtsl2	0.001406925	2.34E-05	-1.108908975
ENSRNOG00000012436	Adh1	0.045827148	0.003320127	1.359357423
ENSRNOG00000001714	Atp13a4	0.013084433	0.000546832	1.158973336
ENSRNOG00000018603	Carns1	0.00535769	0.000158023	-1.05053914
ENSRNOG00000015036	Ctgf	0.008164006	0.000284963	-1.118090634
ENSRNOG00000015085	Dmpk	8.93E-12	6.15E-15	-1.201505113
ENSRNOG00000019914	Fam57b	0.018894887	0.000897275	-1.064498634
ENSRNOG00000045636	Fasn	4.33E-18	1.12E-21	-1.119811881
ENSRNOG00000002800	Gdpd2	0.002125594	4.32E-05	-1.349533655
ENSRNOG00000014835	Il1rl1	0.009719165	0.000361812	1.304648834
ENSRNOG00000006859	Insig1	5.37E-11	4.36E-14	-1.019471233
ENSRNOG00000050240	LOC100909856	0.00104498	1.59E-05	1.101469674
ENSRNOG00000048982	LOC100912282	0.011679484	0.000464901	-1.802966546
ENSRNOG00000005064	LOC103694902	1.18E-06	3.35E-09	1.994999133
ENSRNOG00000058500	LOC103694908	0.005094758	0.000146217	1.423189517
ENSRNOG00000045911	Mepce	0.009255921	0.00033903	8.79750614
ENSRNOG00000001959	Mx1	0.04525132	0.003251049	-1.038250972
ENSRNOG00000001963	Mx2	0.006543494	0.000215428	-1.177500275
ENSRNOG00000007607	Nr4a1	0.030145565	0.001818659	-1.485012061
ENSRNOG00000003348	Rasd1	0.000556056	6.90E-06	-1.237888683
ENSRNOG00000049913	Rbm12	0.006385037	0.000207107	8.045602872
ENSRNOG00000011901	Rrad	0.000964255	1.44E-05	-1.062014779
ENSRNOG00000013552	Scd	3.63E-22	6.26E-26	-1.898943385
ENSRNOG00000046005	Scd2	3.60E-12	2.17E-15	-1.310704862
ENSRNOG00000010210	Slc7a11	0.005071297	0.000144844	1.063420789
ENSRNOG00000003463	Srebf1	1.81E-31	1.56E-35	-1.419819077
ENSRNOG00000005874	Tle2	0.000432315	4.63E-06	-1.110852252
ENSRNOG00000020989	Tm7sf2	2.90E-10	3.25E-13	-1.083553374
ENSRNOG00000052512	Vps37c	2.03E-06	6.14E-09	7.866679894

Table S5. Differentially expressed genes in NRK cells after HBCD exposure				
Gene ID	Gene Name	Q value	P value	Log FC
ENSRNOG00000058555	7SK	0.004253	0.000157	-1.4808
ENSRNOG00000060735	AABR07053136.1	0.019021	0.001311	-1.05129
ENSRNOG00000000967	Aacs	2.22E-13	1.91E-16	-1.07028
ENSRNOG00000019189	Acat2	5.74E-15	3.46E-18	-1.19598
ENSRNOG00000018755	Acss2	2.64E-16	9.11E-20	-1.14304
ENSRNOG00000027742	Adamtsl2	0.001026	2.48E-05	-1.10917
ENSRNOG00000005008	Angpt4	0.000629	1.36E-05	1.010264
ENSRNOG00000014363	Arhgef3	0.001811	5.26E-05	-1.07026
ENSRNOG00000001714	Atp13a4	0.015984	0.001022	1.104248
ENSRNOG00000017073	Car9	4.81E-09	9.54E-12	1.166634
ENSRNOG00000018603	Carns1	0.001931	5.74E-05	-1.12995
ENSRNOG00000006715	Ccr1	0.004696	0.000181	2.147687
ENSRNOG00000036674	Cd7	0.016851	0.001094	-1.34314
ENSRNOG00000049700	Cda	0.014894	0.000918	2.309605
ENSRNOG00000014387	Chac1	1.53E-05	1.19E-07	1.230263
ENSRNOG00000004540	Clec3b	0.000898	2.11E-05	-1.66503
ENSRNOG00000049782	Csf2ra	0.006148	0.000263	-1.06671
ENSRNOG00000046643	Cyp3a9	0.018824	0.001296	1.084142
ENSRNOG00000007234	Cyp51	2.93E-13	2.78E-16	-1.08104
ENSRNOG00000020776	Dhcr7	1.97E-07	7.32E-10	-1.02226
ENSRNOG00000015736	Dhrs3	3.26E-05	3.15E-07	1.39321
ENSRNOG00000018247	Dhx58	7.04E-05	7.64E-07	-1.26691
ENSRNOG00000018029	Doc2g	0.030345	0.002503	-1.02722
ENSRNOG00000015880	Dpep1	0.00307	0.000103	-1.31477
ENSRNOG00000006931	Eepd1	0.000113	1.32E-06	-1.15642
ENSRNOG00000043300	Enho	0.000106	1.21E-06	-1.37133
ENSRNOG00000004089	Enpp2	0.038162	0.00348	-1.45214
ENSRNOG00000024688	Erfe	0.00111	2.71E-05	1.139392
ENSRNOG00000019914	Fam57b	4.99E-05	5.12E-07	-1.69035
ENSRNOG00000043377	Fdps	1.07E-13	8.33E-17	-1.23845
ENSRNOG00000002800	Gdpd2	0.000287	4.70E-06	-1.52088
ENSRNOG00000047697	Ggt1	0.005437	0.000223	1.026872
ENSRNOG00000038328	Gjc2	0.000874	2.03E-05	-1.17115
ENSRNOG00000022321	Hapln3	0.022358	0.00165	-1.2004
ENSRNOG00000016552	Hmgcs1	1.07E-13	8.20E-17	-1.56974
ENSRNOG00000002826	Hsd17b7	1.64E-12	1.84E-15	-1.44926
ENSRNOG00000016690	Idi1	5.88E-11	7.60E-14	-1.24105
ENSRNOG00000026605	Ifi27l2b	2.26E-05	1.81E-07	1.057753
ENSRNOG00000011680	Il16	0.006787	0.000303	-1.18333
ENSRNOG00000014835	Il1rl1	4.99E-05	5.17E-07	1.870023
ENSRNOG00000021217	Itga10	0.000329	5.78E-06	-1.03912

ENSRNOG00000036703	Itgax	4.52E-05	4.59E-07	1.22292
ENSRNOG00000027601	Lhfpl1	0.024057	0.001816	-1.11355
ENSRNOG00000050240	LOC100909856	0.001651	4.74E-05	1.039389
ENSRNOG00000047977	LOC100910163	0.008683	0.000417	-1.18064
ENSRNOG00000020197	LOC100911932	0.00025	3.92E-06	-1.20056
ENSRNOG00000047158	LOC103689947	3.34E-29	2.88E-33	-2.02271
ENSRNOG00000005064	LOC103694902	1.17E-06	5.53E-09	1.966432
ENSRNOG00000058500	LOC103694908	0.000119	1.45E-06	1.808777
ENSRNOG00000046021	LOC108348172	0.039846	0.003716	-1.18297
ENSRNOG00000054549	Lss	1.63E-19	4.22E-23	-1.06333
ENSRNOG00000004610	Lum	3.42E-06	1.94E-08	-1.40207
ENSRNOG00000024620	Mamdc2	0.035725	0.003184	1.908016
ENSRNOG00000049132	Map2k3	0.041097	0.003921	-1.7194
ENSRNOG00000032832	Mmp10	0.017422	0.001143	1.05024
ENSRNOG00000008478	Mmp13	0.021463	0.001558	1.263168
ENSRNOG00000031033	Mt-nd2	0.018037	0.001208	1.006586
ENSRNOG00000013376	Mvd	1.17E-12	1.21E-15	-1.26268
ENSRNOG00000001959	Mx1	0.023311	0.001748	-1.10711
ENSRNOG00000001963	Mx2	0.000377	6.89E-06	-1.43804
ENSRNOG00000018997	Myh7b	9.98E-06	6.71E-08	-1.29873
ENSRNOG00000002215	Mylk	0.012901	0.00073	-1.10159
ENSRNOG00000057814	Nsdhl	3.50E-15	1.81E-18	-1.17161
ENSRNOG00000003018	Olfml2b	2.15E-06	1.21E-08	-2.08002
ENSRNOG00000011136	Osr2	2.52E-05	2.08E-07	-1.06206
ENSRNOG00000039469	Pcdhb19	0.006138	0.000262	-1.48538
ENSRNOG00000012337	Pde1c	5.51E-05	5.79E-07	-1.88049
ENSRNOG00000018859	Pik3ip1	3.31E-10	5.42E-13	-1.14001
ENSRNOG00000013971	Psat1	3.25E-10	5.04E-13	1.134908
ENSRNOG00000005277	Ptprv	1.23E-05	8.71E-08	-1.09778
ENSRNOG00000011901	Rrad	0.001307	3.44E-05	-1.02138
ENSRNOG00000001295	S100b	0.009528	0.000479	-1.73204
ENSRNOG00000013552	Scd	6.80E-20	1.17E-23	-1.80472
ENSRNOG00000010210	Slc7a11	0.004557	0.000173	1.051953
ENSRNOG00000006033	Spon2	1.60E-07	5.66E-10	-1.46022
ENSRNOG00000024028	Sprr1a	0.014773	0.000907	2.249811
ENSRNOG00000047306	Stap2	2.12E-06	1.15E-08	-1.40874
ENSRNOG00000005874	Tle2	0.00033	5.82E-06	-1.10741
ENSRNOG00000020989	Tm7sf2	3.87E-16	1.67E-19	-1.3524
ENSRNOG00000033734	Tnnt2	0.006659	0.000294	-1.032
ENSRNOG00000007319	Trib3	0.000275	4.41E-06	1.08373
ENSRNOG00000003587	Vegfd	0.043734	0.004312	-1.14114

**Table S6**. **Differentially expressed genes in NRK cells after TBBPA exposure**				
Gene ID	Gene Name	Q value	P value	Log FC
ENSRNOG00000018126	Abca1	6.51E-12	1.59E-15	-1.20576
ENSRNOG00000008005	Akna	9.39E-05	4.37E-07	1.043125
ENSRNOG00000009734	Akr1b10	1.19E-16	1.03E-20	1.479955
ENSRNOG00000007546	Asns	1.82E-07	1.73E-10	1.035153
ENSRNOG00000050431	Aspnl1	0.027305	0.000664	-1.09719
ENSRNOG00000001714	Atp13a4	0.002176	1.96E-05	1.484226
ENSRNOG00000050419	Avil	0.000938	6.71E-06	1.291104
ENSRNOG00000033697	Casp4	4.79E-05	1.61E-07	1.29906
ENSRNOG00000014387	Chac1	4.41E-05	1.44E-07	1.268179
ENSRNOG00000019851	Cox6a2	0.043825	0.001312	1.402093
ENSRNOG00000015036	Ctgf	3.63E-05	1.13E-07	-1.7789
ENSRNOG00000015085	Dmpk	2.38E-09	8.19E-13	-1.17659
ENSRNOG00000010805	Fabp4	0.007492	9.75E-05	1.714904
ENSRNOG00000002800	Gdpd2	0.018035	0.000348	-1.25861
ENSRNOG00000026605	Ifi27l2b	6.51E-12	1.68E-15	1.701851
ENSRNOG00000027433	LOC100910708	4.14E-09	1.78E-12	1.577989
ENSRNOG00000031045	LOC100911252	0.029677	0.000747	1.168726
ENSRNOG00000047551	LOC100911615	0.018644	0.000367	-10.0694
ENSRNOG00000048982	LOC100912282	0.000283	1.63E-06	-2.77123
ENSRNOG00000059883	LOC100912578	0.001378	1.10E-05	1.006203
ENSRNOG00000005064	LOC103694902	0.000257	1.46E-06	1.661777
ENSRNOG00000008478	Mmp13	7.41E-06	1.66E-08	2.403855
ENSRNOG00000001963	Mx2	0.042985	0.001267	-1.09432
ENSRNOG00000049695	Myh2	0.014317	0.000222	-1.1604
ENSRNOG00000011136	Osr2	1.78E-05	4.75E-08	-1.18425
ENSRNOG00000019328	Phgdh	5.02E-05	1.73E-07	1.012059
ENSRNOG00000027331	Ppef1	0.002072	1.84E-05	1.392698
ENSRNOG00000013971	Psat1	2.72E-07	3.05E-10	1.025828
ENSRNOG00000015072	Ptgr1	8.60E-09	5.19E-12	1.181869
ENSRNOG00000003348	Rasd1	5.09E-05	1.81E-07	-1.54785
ENSRNOG00000055564	RGD1564664	0.037655	0.001038	-1.00848
ENSRNOG00000016949	Slc25a33	0.004087	4.33E-05	1.051265
ENSRNOG00000010210	Slc7a11	6.65E-08	5.15E-11	1.944944
ENSRNOG00000002218	Stbd1	8.97E-05	4.02E-07	1.316929
ENSRNOG00000017628	Tagln	0.025161	0.000581	-1.14729
ENSRNOG00000007319	Trib3	1.73E-06	3.05E-09	1.467291
ENSRNOG00000017854	Ucp2	0.0067	8.66E-05	1.335377
ENSRNOG00000052512	Vps37c	0.005346	6.16E-05	5.670153

**Table S7. Top 10 BDE 47-enriched gene sets in HK-2 cells according to Gene Set Enrichment Analysis**						
Name	Parent Folder	# of Entities	# of Measured Entities	Measured Entities	Median Change	p-value
Eicosanoids in Inflammation	Inflammatory Mediators of Systemic Reaction; Inflammatory Mediators of Systemic Reaction (Inflammation Pathways); generic; generic	47	8	ALOX5;PLA2G4B;PLA2G4C;GGT1;PTGES2;GGT7;PLA2G4A;ALOX5AP	1.30	0.00
Kynurenine Metabolites in T-Cell Apoptosis	Aryl Hydrocarbon Receptor Signaling; Aryl Hydrocarbon Receptor Signaling (Receptor Signaling); T-cell Activation and Differentiation; lymphatic system; mitochondria	26	7	ACMSD;QPRT;KYAT1;AFMID;AADAT;EIF2AK4;KYNU	1.21	0.02
Coagulation Cascade	Inflammation Initiation; Inflammation Initiation (Inflammation Pathways); blood; hematological system	38	8	F12;F3;PROS1;VKORC1;COL1A1;TFPI;THBD;PLAT	1.17	0.03
Glutamate Release	Neurotransmitter Release Cycles; Neurotransmitter Release Cycles (Nociception Pathways); nerve tissue; nervous system; secretory vesicle	23	9	SLC38A3;GLS2;SLC1A5;GLUL;SLC38A1;GLS;SLC1A1;SLC7A6;SLC1A3	-1.16	0.01
Vitamin A (Retinol) Metabolism and Visual Cycle	Cofactors and Vitamins Metabolism; Cofactors and Vitamins Metabolism (Metabolic Pathway); Vitamins Biology; generic; generic	93	18	CYP1A1;CYP26A1;RDH5;CYP3A5;CYP26C1;CYP1B1;ALDH1A3;BCO1;DHRS4;RETSAT;DGAT1;DGAT2;LRAT;ADH5;CYP2J2;RDH11;CYP2S1;AOX1	1.15	0.01
Lipoxins and Resolvins in Inflammation Resolution	Inflammation Resolution; Inflammation Resolution	48	9	ALOX5;HPGD;LTB4R;CX3CL1;LTA4H;GPR1	1.15	0.00

	(Inflammation Pathways); generic; generic			32;SELL;TNF;P2RY2		
rRNA Transcription and Processing	RNA Transcription; RNA Transcription (Cell Process); generic; generic; nucleus	35	124	RPL13A;RPS19;RPS23;RPS27A;FBL;RPL34;RPL13;RPS9;RPS3;RPS12;RPS21;RPS14;RPL41;RPL28;RPL21;RPS3A;RPS16;RPL37A;RPL26;RPL36A;RPS10;RPS28;RPS5;RPL37;RPS20;RPS29;RPS6;RPS13;RPL7A;RMRP;RPL27A;GTF3C6;RPL17;RPL18;RPL10A;RPL22;RPS25;RPS27;RPS18;FAU;RPL24;RPL31;RPP21;RPS2;RPS24;RPSA;RPL9;RPL3;RPL7;RPLP0;POLR1D;RPL36;POP5;RPL8;RPL4;RPL35A;RPS17;RPL30;UBA52;RPS4Y1;TTF1;RPL23A;RPS7;RPS15A;RPL15;RPS8;RPL38;RPL14;RPL18A;RPS15;RPL19;RPL10;POLR3K;MAF1;RPL39;POLR3GL;RPS11;RPL5;RPL11;RPL6;TAF1C;RACK1;RPL27;RPL35;RPL32;BRF1;RPP14;CDK1;RPP38;GTF3C5;POP4;RPL23;POLR3H;RPS26;RPL29;RPP30;BRF2;POLR1E;POLR1C;TAF1B;DR1;UBTF;POLR3C;POLR3D;POLR3F;GTF3A;POLR3E;TBP;GTF3C2;RB1;CD3EAP;TOP1;RPP40;GTF3C1;TAF1A;GTF3C3;POLR3A;BDP1;GTF3C4;POLR3G;POLR1B;POP1;POLR1A;POLR3B	1.15	0.00
Translation	RNA Machinery;	84	146	RPL13A;RPS19;RPS23;RPS2	1.14	0.00

	RNA Machinery (Cell Process); endoplasmic reticulum; generic; generic			7A;RPL34;RPL13;RPS9;RPS3;RPS12;RPS21;RPS14;RPL41;RPL28;RPL21;RPS3A;RPS16;RPL37A;RPL26;RPL36A;RPS10;RPS28;RPS5;TPT1;EIF3K;RPL37;RPS20;PTRHD1;RPS29;RPS6;RPS13;RPL7A;EEF1A1;PTRH1;RPL27A;EEF1D;RPL17;RPL18;RPL10A;RPL22;RPS25;RPS27;RPS18;FAU;RPL24;RPL31;RPS2;RPS24;RPSA;EEF1G;RPL9;RPL3;EIF3F;RPL7;RPLP0;EEF1A2;RPL36;QARS;RPL8;RPL4;EEF1B2;RPL35A;RPS17;RPL30;UBA52;RPS4Y1;RPL23A;RPS7;RPS15A;RPL15;RPS8;RPL38;EIF1AY;RPL14;RPL18A;RPS15;RPL19;RPL10;EIF2S2;RPL39;RPS11;RPL5;RPL11;EIF1;RPL6;RACK1;RAMAC;RPS6KA1;RPL27;EIF3L;RPL35;FARS2;RPL32;EIF3I;EIF3G;EIF5B;RPS6KB2;EIF3E;EIF3M;RPL23;EIF3H;RPS26;RPL29;DARS;PTRH2;EEF2K;RARS;PABPC1;EIF2S3;SARS;KTN1;EIF3D;AARS;EEF2;EIF3CL;EIF3A;EIF4E;NARS;FARSA;LARS2;MRPL58;EIF1AX;EIF3B;GARS;VARS;GSPT1;EIF4A1;EIF3C;CARS;HARS;LARS;ETF1;EIF2S1;RNGTT;MARS;EIF3J;RNMT;TARS;EIF4G1;YARS;IARS;EPRS;KARS;RPS6KB		

				1;WARS;MARS2;ABCE1		
Leukocyte Adhesion to Endothelial Cell	Leukocyte Adhesion; Leukocyte Adhesion (Immunological Pathways); lymphatic system	41	15	CD99;F11R;ICAM2;ICAM3;CD58;GLG1;NECTIN2;JAM3;SELL;VCAM1;ICAM1;ITGB1;PVR;ITGA4;ITGB2	-1.14	0.04
Fatty Acids Cause Oxidative Stress and Mitochondria Decline in Aging	Organelle Instability Associated with Aging; Organelle Instability Associated with Aging (Aging Biology); generic; generic; mitochondria	41	34	PLA2G4B;PPARGC1A;PLA2G4C;UQCRQ;NDUFV1;GPX4;UQCRB;SLC27A1;NQO1;ATP6V1E1;GPX1;ATP5F1D;ATP5PO;UQCRFS1;ATP6V1F;UQCRC1;ATP6V1A;ATP6V1H;NDUFV2;ATP6V0E1;PLA2G12A;ATP6V0A4;PPARA;ATP6V1D;ATP6AP1;ATP6V1B2;SREBF1;UQCRC2;UCP2;ATP6V0A1;NFE2L2;SLC27A4;PLA2G4A;LDLR	1.13	0.02

Table S8. Top 10 HBCD-enriched gene sets in HK-2 cells according to Gene Set Enrichment Analysis

Name	Parent Folder	# of Entities	# of Measured Entities	Measured Entities	Median Change	p-value
Eicosanoids in Inflammation	Inflammatory Mediators of Systemic Reaction; Inflammatory Mediators of Systemic Reaction (Inflammation Pathways); generic; generic	47	8	PLA2G4B;PLA2G4C;ALOX5;ALOX5AP;GGT1;GGT7;PTGES2;PLA2G4A	1.335009	0.004971
Gastric and Pancreatic Lipase Function	Lipogenesis and Lipolysis; Lipogenesis and Lipolysis (Biological Process); digestive system	19	7	TGFA;MGLL;HES1;PPARG;SLC27A1;EGF;GATA6	1.260558	0.02448
CCR1 Expression Targets	Chemokine GPCRs; GCPR Family; GCPR Family (Nociception Receptors Signaling); nerve tissue; nervous system	28	8	MMP9;TNF;MMP1;VEGFA;ICAM1;STAT3;JAK1;STAT1	1.208391	0.000748
S-cell: Secretin Production	Cells of Gastrointestinal Truct Activation; Cells of Gastrointestinal Truct Activation (Eating Behavior and Metabolism Regulation); digestive system; epithelium	11	15	ASIC3;MME;PLB1;TMEM37;DPP4;PLAAT3;ABHD3;ASIC1;JMJD7;GRIN2B;TMCO1;CUL5;TRPM4;GRIN1;CHRNA10	1.178122	0.001394
Coagulation Cascade	Inflammation Initiation; Inflammation Initiation (Inflammation Pathways); blood; hematological system	38	8	THBD;F3;VKORC1;PROS1;F12;TFPI;COL1A1;PLAT	1.153841	0.025038
Glycolysis	Eating Behavior and Metabolism Regulation; Eating Behavior and Metabolism Regulation (Biological Process); generic; generic	36	13	ENO2;PGK1;HK2;ALDOA;HK1;ENO1;TPI1;GPI;GAPDH;PGAM1;ENO3;PKM;PFKM	1.148631	0.003758

Insulin Influence on Lipogenesis	Insulin Release and Action; Insulin Release and Action (Eating Behavior and Metabolism Regulation); generic	43	25	APOA2;IRS2;PLPP5;SLC27A1;SLC2A1;ACSL1;GK;SLC2A4;PLPP2;APOC1;APOE;GPAT3;PLPP4;PLPP3;PLPPR2;INSR;GK5;DGAT1;PLPP1;AKT1;TBC1D4;DGAT2;GPD1L;PDPK1;IRS1	1.139717	0.005377
Keep out Signal: LTF Inhibits Neutrophil Recruitment and Inflammation	Efferocytosis; Efferocytosis (Inflammation Resolution); oncogenic; oncogenic	18	8	C5AR1;TNF;CSF2;RPS19;CEBPA;REL;CXCL8;IL6	1.12399	0.00257
Vitamin A (Retinol) Metabolism and Visual Cycle	Cofactors and Vitamins Metabolism; Cofactors and Vitamins Metabolism (Metabolic Pathway); Vitamins Biology; generic; generic	93	18	CYP1A1;CYP26A1;RDH5;CYP26C1;CYP3A5;RETSAT;LRAT;RDH11;BCO1;AOX1;DGAT1;CYP2S1;DHRS4;DGAT2;ADH5;CYP2J2;ALDH1A3;CYP1B1	1.10904	0.026023
Find me Signal: Apoptotic Cell Attracts Phagocyte	Efferocytosis; Efferocytosis (Inflammation Resolution); oncogenic; oncogenic; secretory vesicle	25	16	ADAM17;RPS19;YARS;ABCA1;S1PR2;CX3CL1;AIMP1;CASP7;ADAM10;S1PR5;P2RY2;ANXA1;PANX1;CXCL8;S1PR1;THBS1	-1.10252	0.011763

**Table S9. Top 10 TBBPA-enriched gene sets in HK-2 cells according to Gene Set Enrichment Analysis**						
**Name**	Parent Folder	# of Entities	# of Measured Entities	Measured Entities	Median Change	p-value
Eicosanoids in Inflammation	Inflammatory Mediators of Systemic Reaction; Inflammatory Mediators of Systemic Reaction (Inflammation Pathways); generic; generic	47	8	ALOX5AP;PLA2G4C;PLA2G4B;PLA2G4A;PTGES2;ALOX5;GGT7;GGT1	1.345669	0.003006
Keep out Signal: LTF Inhibits Neutrophil Recruitment and Inflammation	Efferocytosis; Efferocytosis (Inflammation Resolution); oncogenic; oncogenic	18	8	C5AR1;CXCL8;IL6;RPS19;CSF2;CEBPA;TNF;REL	1.340272	0.023219
Glycolysis	Eating Behavior and Metabolism Regulation; Eating Behavior and Metabolism Regulation (Biological Process); generic; generic	36	13	ENO3;PFKM;HK2;GAPDH;HK1;TPI1;PKM;ENO1;ENO2;GPI;ALDOA;PGAM1;PGK1	-1.31609	0.001697
Complement Alternative Pathway	Complement Activation; Complement Activation (Biological Process); generic; hematological system; plasma membrane	30	6	C5AR1;C5;CFP;CFB;CFH;CFI	1.289772	0.021657
Glutamate Release	Neurotransmitter Release Cycles; Neurotransmitter Release Cycles (Nociception Pathways); nerve tissue; nervous system; secretory vesicle	23	9	SLC38A3;SLC1A5;GLS2;SLC38A1;SLC1A3;SLC1A1;SLC7A6;GLUL;GLS	-1.28075	0.001096
RHO Signaling (Light Signal Termination and Photoreceptor Recovery)	Visual Phototransduction; Visual Phototransduction (Biological Process); retina; sensory system	30	12	PLCB1;PRKCB;PRKCA;SLC24A1;PLCXD1;RGS9;PLCH1;PDE6B;PLCXD2;PLCE1;PLCB3;PLCB4	1.218748	0.013279
Chromosome Condensation	Cell Division; Cell Division (Cell Process);	21	20	STK4;H3F3B;STAG2;STAG1;NCAPH2;AUR	-1.20377	0.02469

	chromosome; generic; generic; nucleus			KB;SMC2;PARP1;AURKA;RAD21;SMC4;NCAPG2;CDK1;NCAPG;PLK1;NCAPD3;NCAPD2;ESPL1;TOP2A;NCAPH		
Male Sex Determination	Reproductive Biology; Reproductive Biology (Biological Process); reproductive system	38	11	ZFPM2;PTGDS;FOXL2;SP1;SIX4;SIX1;AMH;CITED2;VNN1;CBX2;SOX9	-1.16044	0.043752
GABA Release	Neurotransmitter Release Cycles; Neurotransmitter Release Cycles (Nociception Pathways); nerve tissue; nervous system; secretory vesicle	28	23	GPT2;SLC38A3;STX1B;STX8;STX1A;STX16;CSAD;SLC1A5;STX4;GLS2;SLC38A1;STX18;STX5;STX12;STX17;STX6;STX10;STX3;STX7;STX2;SLC7A6;GLUL;GLS	1.141516	0.020208
Macrophage Related Iron Uptake and Release	Monocytes Activation; Monocytes Activation (Myeloid Cells Activation in Inflammation); generic; hematological system; secretory vesicle	39	15	HMOX1;CEBPB;FTH1;FTL;IL6;NFKB2;ACO1;CSF2;SLC11A2;HIF1A;TNF;SLC40A1;IREB2;HMOX2;TFRC	1.139328	0.014949

**Table S10. Top 10 BDE 47-enriched gene sets in NRK cells according to Gene Set Enrichment Analysis**						
**Name**	Parent Folder	# of Entities	# of Measured Entities	Measured Entities	Median Change	p-value
Estrogen Deficiency in Female Obesity	Aging Related Diseases; Obesity; Obesity (Endocrine and Metabolic Diseases); adipose tissue; connective tissue; endocrine system	16	9	PRKAA1;PRKAA2;PPARG;LPL;ACACA;SLC2A4;FASN;SREBF1;SCD	-1.48779	0.001428
Coagulation Cascade	Inflammation Initiation; Inflammation Initiation (Inflammation Pathways); blood; hematological system	38	7	TFPI;VKORC1;PLAT;COL1A1;PROS1;COL1A2;F3	-1.32108	0.018073
Single-Strand Mismatch	DNA Repair; DNA Repair (Cell Process); generic; generic; nucleus	19	25	POLE;EXO1;RFC5;POLD2;RPA2;RFC4;RFC3;LIG1;RFC2;PCNA;FEN1;POLD1;MSH2;MLH1;RPA1;MSH3;E2F6;POLD3;MSH6;RFC1;RPA3;MLH3;PURB;PMS1;POLD4	1.273773	8.36E-07
Chromosome Condensation	Cell Division; Cell Division (Cell Process); chromosome; generic; generic; nucleus	21	23	CASP4;NCAPD3;PLK1;CDK1;AURKB;NCAPG;SMC2;TOP2A;ESPL1;AURKA;SMC4;NCAPG2;NCAPD2;RAD21;NCAPH2;PARP1;HIST1H3A;STK4;CASP1;STAG1;STAG2;HIST1H3E;HIST1H3H	1.270261	0.001954
Protein Nuclear Import and Export	Proteins Turnover; Proteins Turnover (Cell Process); generic; generic; nucleus	26	37	NUP43;NUP85;NDC1;NXT1;NUP205;NUTF2;RANBP1;NUP35;KPNA2;NUP153;NUP93;CSE1L;NUP58;NUP54;RAN;KPNA1;POM121;RCC1;NUP155;NUP50;KPNB1;NUP133;GLE1;NUP37;NUP98;RANGAP1;AHCTF1;XPO1;XPO4;TNPO1;NUP42;RAE1;XPO6;XPO7;X	1.218144	4.03E-07

				PO5;TNPO3;NXF1		
Complement Classical Pathway	Complement Activation; Complement Activation (Biological Process); generic; hematological system; plasma membrane	28	5	C1QBP;C5AR2;C2;C1R;C1S	-1.19024	0.028552
Extracellular Matrix Turnover	Cellular Contacts; Cellular Contacts (Cell Process); generic; generic; plasma membrane	36	29	MMP13;DYM;LRP1;MMP16;FN1;MMP2;COL6A3;COL6A2;ECM1;MMP19;TIMP1;COL6A1;MMP14;TIMP2;COL5A3;MMP10;RECK;COL5A2;MMP28;COL1A1;COL1A2;COL5A1;ADAMTS1;THBS2;COL4A2;MMP24;MMP15;COL4A1;MMP11	-1.18661	0.001458
Lipids Enhance Apoptotic Cell Engulfment and Reduce Inflammation	Efferocytosis; Efferocytosis (Inflammation Resolution); oncogenic; oncogenic; secretory vesicle	36	17	C1QBP;RARA;RXRA;NR1H2;RXRB;RARG;MFGE8;CD14;PPARG;NFKB2;IL6;TGM2;PPARD;MERTK;ABCA1;SREBF1;NR4A1	-1.17878	0.000314
Insulin Secretion	Insulin Release and Action; Insulin Release and Action (Eating Behavior and Metabolism Regulation); Pancreatic Beta-cells in Diabetes; endocrine system	38	11	SLC2A1;CREB1;MTPN;PLCB1;PLCB3;ITPR1;PLCB4;SYTL4;ACLY;GIP;PLCL1	-1.17784	0.044514
Double Strand DNA Homologous Repair	DNA Repair; DNA Repair (Cell Process); generic; generic; nucleus	30	37	POLE;POLE2;XRCC3;BRCA2;BRCA1;BARD1;POLD2;RAD51;XRCC2;POLA1;LIG1;H2AFX;ATM;POLD1;ABRAXAS1;MDC1;RBBP8;POLH;POLE3;UBE2N;REV3L;MRE11;RAD50;SMC1A;BRCC3;POLK;POLB;LIG3;NBN;UIMC1;POLI;ATRX;POLE4;POLM;RAD52;POLL;LIG4	1.175153	3.64E-05

Table S11. Top 10 HBCD-enriched gene sets in NRK cells according to Gene Set Enrichment Analysis

**Name**	Parent Folder	# of Entities	# of Measured Entities	Measured Entities	Median Change	p-value
Estrogen Deficiency in Female Obesity	Aging Related Diseases; Obesity; Obesity (Endocrine and Metabolic Diseases); adipose tissue; connective tissue; endocrine system	16	9	PRKAA2;PRKAA1;PPARG;ACACA;SREBF1;SLC2A4;FASN;LPL;SCD	-1.4985	7.93E-05
Complement Cascade Activation by Pentraxins	Systemic Effects in Inflammation; Systemic Effects in Inflammation (Inflammation Pathways); blood; hematological system	29	5	FCGR2A;SNRNP70;C2;CFH;C1S	-1.29582	0.003759
Complement Classical Pathway	Complement Activation; Complement Activation (Biological Process); generic; hematological system; plasma membrane	28	5	C1QBP;C5AR2;C1R;C2;C1S	-1.26217	0.029818
Gastric and Pancreatic Lipase Function	Lipogenesis and Lipolysis; Lipogenesis and Lipolysis (Biological Process); digestive system	19	6	TGFA;GATA6;PPARG;SLC27A1;HES1;GIP	-1.24188	0.0082
Single-Strand Mismatch	DNA Repair; DNA Repair (Cell Process); generic; generic; nucleus	19	25	POLE;EXO1;POLD2;RFC3;RPA2;RFC5;LIG1;FEN1;MLH1;POLD1;RFC4;RFC2;POLD3;MSH3;MSH6;RPA1;PCNA;MSH2;E2F6;PURB;RFC1;MLH3;RPA3;PMS1;POLD4	1.225979	0.000223
Complement Activation in Macular Degeneration	Age-Related Macular Degeneration; Age-Related Macular Degeneration (Ophthalmology/Eye Diseases); Aging Related Diseases; sensory system	45	11	CD55;C1QBP;HTRA1;MASP2;CD46;CLU;CFP;C1R;C2;CFH;C1S	-1.22363	0.006652

Coagulation Cascade	Inflammation Initiation; Inflammation Initiation (Inflammation Pathways); blood; hematological system	38	7	TFPI;VKORC1;COL1A1;COL1A2;F3;PLAT;PROS1	-1.22245	0.037605
Glycolysis	Eating Behavior and Metabolism Regulation; Eating Behavior and Metabolism Regulation (Biological Process); generic; generic	36	15	HK3;PGK1;ENO1;GAPDH;PGAM1;PKM;TPI1;HK2;ENO2;ALDOA;GPI;ENO3;HK1;PGAM2;PFKM	1.218215	0.0089
Extracellular Matrix Turnover	Cellular Contacts; Cellular Contacts (Cell Process); generic; generic; plasma membrane	36	29	MMP13;MMP10;DYM;LRP1;MMP16;COL6A3;MMP19;RECK;COL6A2;FN1;TIMP1;COL5A2;MMP14;COL1A1;COL5A1;ECM1;COL1A2;MMP2;COL6A1;COL4A2;TIMP2;THBS2;COL5A3;ADAMTS1;COL4A1;MMP24;MMP28;MMP15;MMP11	-1.21317	2.34E-05
Vitamin K Metabolism	Cofactors and Vitamins Metabolism; Cofactors and Vitamins Metabolism (Metabolic Pathway); Vitamins Biology; generic; generic	36	11	PRRG4;PRRG1;VKORC1L1;GGCX;VKORC1;PRRG2;F3;NQO1;MGP;PROS1;GAS6	-1.20217	0.031923

Table S12. Top 10 TBBPA-enriched gene sets in NRK cells according to Gene Set Enrichment Analysis

Name	Parent Folder	# of Entities	# of Measured Entities	Measured Entities	Median Change	p-value
Estrogen Deficiency in Female Obesity	Aging Related Diseases; Obesity; Obesity (Endocrine and Metabolic Diseases); adipose tissue; connective tissue; endocrine system	16	9	PPARG;PRKAA1;PRKAA2;SREBF1;ACACA;FASN;LPL;SLC2A4;SCD	-1.31323	0.001513
Extracellular Matrix Turnover	Cellular Contacts; Cellular Contacts (Cell Process); generic; generic; plasma membrane	36	29	MMP13;MMP10;DYM;MMP19;MMP16;ECM1;ADAMTS1;MMP14;COL6A2;COL5A3;TIMP1;FN1;LRP1;COL6A1;MMP28;MMP24;COL6A3;TIMP2;MMP2;RECK;COL5A2;MMP15;COL4A2;COL1A2;COL5A1;COL1A1;COL4A1;THBS2;MMP11	-1.25828	2.04E-07
Coagulation Cascade	Inflammation Initiation; Inflammation Initiation (Inflammation Pathways); blood; hematological system	38	7	TFPI;VKORC1;PLAT;PROS1;F3;COL1A2;COL1A1	-1.22793	0.025671
Single-Strand Mismatch	DNA Repair; DNA Repair (Cell Process); generic; generic; nucleus	19	25	EXO1;RPA2;RFC5;POLD2;RFC4;RFC3;RFC2;PCNA;POLE;MLH3;RPA3;FEN1;LIG1;POLD3;MLH1;E2F6;MSH3;POLD1;MSH2;MSH6;RPA1;PMS1;RFC1;POLD4;PURB	1.171065	0.007218
Vasodilation Activation	Vascular System Response; Vascular System Response (Inflammation Pathways); cardiovascular system	28	14	ADCY8;ADCY3;PPP1CB;PRKACA;ADCY7;CALML4;PRKACB;ADCY9;CACNA1C;ADCY6;m_Calm1;PTGIR;ADRB2;MYLK	-1.16983	0.000138
GRM1/5 (Postsynaptic) -> Ion Channels	Glutamate Receptors; Glutamate Receptors; Glutamate Receptors	35	18	ADCY8;ADCY3;PLCB3;PLCB1;CACNA1B;PRKACA;ADCY7;GNAQ;PRKACB;ADCY9;ADC	-1.16983	0.000246

	(Ligand Gated Ion Channels); nerve tissue; nervous system			Y6;CACNA1A; PRKCE;TRPV2 ;PLCB4;ITPR1; PLCL1;KCNJ9		
Translation	RNA Machinery; RNA Machinery (Cell Process); endoplasmic reticulum; generic; generic	84	129	WARS;EPRS; RPS27;RPL36; YARS;CARS;R PL31;RPL17;S ARS;NARS;GA RS;RPL35A;R PS6KA1;AARS ;RPL27A;MAR S;RPS19;RPL2 4;RPS8;IARS; EIF2S2;RPS11 ;RPL35;RPL39; RPL37;RPS15 A;RPS24;RPS 25;EIF4A1;TA RS;RPL30;RP S7;VARS;RPS 18;RPL15;LAR S;EIF2S1;ABC E1;PTRHD1;m _Rps3a1;EIF3 C;FARS2;RPL 7A;MRPL58;R PL13;RPS27A; RPS6;RPL23A; RPL27;RPS3A; RPL11;PTRH2; RPS16;RPS21; EEF1B2;EIF3D ;RPS10;EIF3G; RPL10A;RPL2 8;RACK1;RPL1 4;EIF3H;RAMA C;RPS3;EIF4E; RPL26;RPL4;R PS6KB1;RPL2 3;RPL10;EIF1A X;RPL5;RPS14 ;RPL22;RPL6; FAU;RNMT;TP T1;EIF3J;RPS5 ;RPL19;RPL18 A;ETF1;EEF1D ;RPL18;RPL37 A;RPL7;RPLP0 ;RARS;RPSA; RPS23;RPS4Y 1;RPL9;FARSA ;EIF1;KARS;D ARS;RPS15;G SPT1;MARS2; EIF3B;RPS12; RPL21;PTRH1; RPS29;EEF1G ;EIF3K;RPL13 A;RPS6KB2;EI F1AY;EIF4G1; RPS28;PABPC 1;EIF3M;RPL3; EIF2S3;EEF1A 1;EIF3A;EIF3F; RPS2;HARS;E EF2K;EEF2;EI F3E;LARS2;RP	1.166574	5.47E-11

				S20;EIF5B;RNGTT		
HRH1/2 -> Vascular Motility	Amine GPCRs; Histamine Receptors/Amine GCPRs; Histamine Receptors/Amine GCPRs (GCPR Family); cardiovascular system; cytoskeleton	28	17	ADCY8;ADCY3;PLCB3;PPP1CB;CACNA1B;PRKACA;ADCY7;CALML4;GNAQ;PRKACB;ADCY9;CACNA1C;NOS3;ADCY6;m_Calm1;ITPR1;MYLK	-1.15566	0.001385
TACR1 -> Membrane Transport	Peptide GPCRs; Tachykinin Receptor/Peptide GCPRs; Tachykinin Receptor/Peptide GCPRs (GCPR Family); generic; generic; plasma membrane	20	15	ADCY8;ADCY3;PRKCA;PLCB3;PLCB1;PRKACA;ADCY7;GNAQ;PRKACB;ADCY9;ADCY6;PLCB4;ITPR1;PLCL1;TACR1	-1.15566	0.003407
PTGFR -> Vasoconstriction	Lipid Like GPCRs; Prostaglandin Receptors/Lipid Like GCPRs; Prostaglandin Receptors/Lipid Like GCPRs (GCPR Family); cardiovascular system; cytoskeleton	15	9	PRKCA;PLCB1;PPP1CB;CALML4;GNAQ;m_Calm1;PTGFR;ITPR1;MYLK	-1.15566	0.036924
